# Synergistic Transcriptional and Post-Transcriptional Regulation of ESC Characteristics by Core Pluripotency Transcription Factors in Protein-Protein Interaction Networks

**DOI:** 10.1371/journal.pone.0105180

**Published:** 2014-08-29

**Authors:** Leijie Li, Liangcai Zhang, Guiyou Liu, Rennan Feng, Yongshuai Jiang, Lei Yang, Shihua Zhang, Mingzhi Liao, Jinlian Hua

**Affiliations:** 1 College of Life Sciences, Northwest A&F University, Yangling, Shaanxi, China; 2 College of Bioinformatics Science and Technology, Harbin Medical University, Harbin, China; 3 Department of Statistics, Rice University, Houston, TX, United States of America; 4 Genome Analysis Laboratory, Tianjin Institute of Industrial Biotechnology, Chinese Academy of Sciences, Tianjin, China; 5 Department of Nutrition and Food Hygiene, School of Public Health, Harbin Medical University, Harbin, China; 6 Department of Biostatistics, School of Science, Anhui Agricultural University, Hefei, China; 7 College of Veterinary Medicine, Shaanxi Centre of Stem Cells Engineering & Technology, Northwest A&F University, Yangling, Shaanxi, China; Scuola Superiore Sant'Anna, Italy

## Abstract

The molecular mechanism that maintains the pluripotency of embryonic stem cells (ESCs) is not well understood but may be reflected in complex biological networks. However, there have been few studies on the effects of transcriptional and post-transcriptional regulation during the development of ESCs from the perspective of computational systems biology. In this study, we analyzed the topological properties of the “core” pluripotency transcription factors (TFs) OCT4, SOX2 and NANOG in protein-protein interaction networks (PPINs). Further, we identified synergistic interactions between these TFs and microRNAs (miRNAs) in PPINs during ESC development. Results show that there were significant differences in centrality characters between TF-targets and non-TF-targets in PPINs. We also found that there was consistent regulation of multiple “core” pluripotency TFs. Based on the analysis of shortest path length, we found that the module properties were not only within the targets regulated by common or multiple “core” pluripotency TFs but also between the groups of targets regulated by different TFs. Finally, we identified synergistic regulation of these TFs and miRNAs. In summary, the synergistic effects of “core” pluripotency TFs and miRNAs were analyzed using computational methods in both human and mouse PPINs.

## Introduction

The capacity to differentiate into different cell types, a property known as pluripotency, is a defining property of embryonic stem cells (ESCs). ESCs are derived from the inner cell mass of the mammalian blastocyst [1], [2]. Pluripotency may be conferred on somatic cells following their fusion with ESCs [3]. During this process, the transcription factor (TF) NANOG is specifically expressed, and this may facilitate fusion-induced pluripotency [4]. Moreover, human and mouse fibroblasts can be reprogrammed into ES-like cells which are called induced pluripotent stem cells (iPS) by forced expression of other TFs (OCT4, SOX2, Klf4, and c-Myc) [5]–[7]. The quality of iPS is enhanced upon selection of cells that express endogenous OCT4 or NANOG [8], [9]. Recently, Deng *et al*. reprogramed somatic cells into pluripotent cells using a combination of seven small-molecule compounds and called them CiPS [10]. Epigenetic modifications (DNA methylation, histone modification, miRNAs and other methods of epigenetic regulation) have also been found to play important roles in the maintenance of ‘stemness’ [11]–[13]. These results indicate that in addition to the genetic factors affecting the maintenance of pluripotency, complex epigenetic factors are also involved in the transformation of ESCs. In order to understand the mechanism by which pluripotency is established and maintained in ESCs, further effort will be required to research all aspects of the properties of molecules and their complex interactions in the biological networks which are involved in transcriptional and post-transcriptional regulation.

According to previous studies, a small set of TFs, including OCT4, SOX2 and NANOG comprise the “core” pluripotency factors in ESCs [14]. OCT4 has long been considered to play essential roles in maintaining pluripotency *in vivo* and *in vitro* [15]. In fact, the concentration of OCT4 is crucial for pluripotency: reduced expression evokes trophoectoderm development, whereas enhanced expression leads to primitive endoderm differentiation [16]. As a transcriptional partner of OCT4, SOX2 assembles on regulatory elements of target genes together with OCT4 to collaborate in transcriptional control, without directly interacting with OCT4 protein [17]. The function of NANOG is to promote the self-renewal of ESCs and alleviate the requirement for Leukemia Inhibitory Factor (LIF) [18], [19]. Among OCT4 targets, about half are associated with SOX2. Furthermore, more than 90% of the target genes shared by OCT4 and SOX2 are also associated with NANOG [20]. Based on the above results, some researchers have constructed biological networks that involve these TFs, and analyzed their properties during the development of ESCs [21]–[24]. In a word, as key factors in the maintenance of the pluripotency and self-renewal of ESCs, OCT4, SOX2 and NANOG coordinate the regulation of downstream genes.

Besides the traditional genetic impacts on the maintenance of ESC pluripotency, epigenetic regulation is also involved in the process of ESC development. In particular, more and more studies have found that miRNAs play important roles during the development of ESCs [25]–[27]. miRNAs are endogenous single strand non-coding RNAs which can inhibit target mRNA expression in a post-transcriptional manner [28]. It is characteristic of miRNAs that they regulate target genes in a minor manner and show temporal and spatial specificity. They may form a complex interaction network with other biological molecules *in vivo*. However it is not clear how the target genes of “core” pluripotency TFs regulate ESC development synergistically with miRNAs.

In this study, we identified protein-protein interaction networks (PPINs) and analyzed the topological properties of the target genes of OCT4, SOX2 and NANOG in human and mouse ESCs. Further, we explored the effects of miRNAs on the post-transcriptional regulation of the target genes of these three “core” pluripotency TFs. We found that the centrality of “core” pluripotency transcription factor target genes is higher than that of randomly selected genes in PPINs. Furthermore, when genes are regulated by more “core” pluripotency TFs, they show more properties of centrality. The target genes regulated by both transcriptional and post-transcriptional methods also have higher centrality properties in PPINs. These results indicate that there are both the complex interactions between different “core” pluripotency TFs during ESC development within transcriptional levels and the interactions occur across both transcriptional and post-transcriptional levels in biological networks.

## Materials and Methods

### Dataset of transcription factor targets

**Figure 1:**
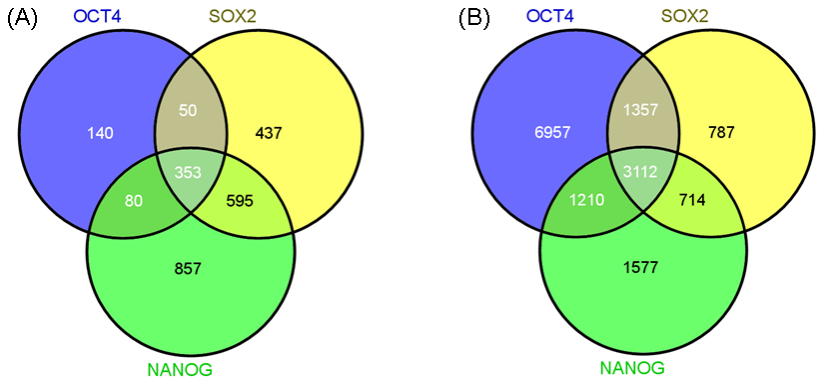
Venn diagram showing the targets of “core” pluripotency TFs. (A) Human targets of TFs. (B) Mouse targets of TFs.

In order to obtain comprehensive target datasets of “core” pluripotency TFs in ESCs, we manually collected related articles in PubMed. Finally, 10 articles and their corresponding datasets were extracted and used in this study (Table 1). The human database contained 3,949 entries, including 623 targets of OCT4, 1,436 targets of SOX2 and 1,886 targets of NANOG (Figure 1 left). The mouse database contained 25,222 entries, including 12,637 targets of OCT4, 5,971 targets of SOX2 and 6,614 targets of NANOG (Figure 1 right). The detailed targets of these three TFs are shown in Table S1 and Table S2.

**Table 1:** Targets of “core” pluripotency TFs in ESCs.

Species	Years	PubMed ID	OCT4-targets	SOX2-targets	NANOG-targets
human	2005	16153702 [20]	623	1279	1687
human	2008	18443585 [46]	0	734	988
mouse	2005	16518401 [47]	778	0	1027
mouse	2008	18555785 [48]	4369	2941	2612
mouse	2008	18358816 [21]	0	819	1284
mouse	2008	18692474 [25]	4320	3380	3114
mouse	2008	18347094 [49]	2151	0	1936
mouse	2010	20362542 [50]	10	10	12
mouse	2011	21477851 [51]	7518	0	0
mouse	2013	23582322 [52]	4417	1699	2492

### Datasets of Protein-Protein interactions

In order to avoid any bias due to the data source, protein-protein interaction data were also downloaded from two different databases: the Biological General Repository for Interaction Datasets (BioGRID) version 3.2.110 (http://theBioGRID.org/) and the Human Protein Reference Database (HPRD) (http://www.hprd.org/) [29], [30]. We then removed duplicated edges and selfloops using Cytoscape and analyzed topological properties using the NetworkAnalyzer tools in Cytoscape [31]–[33]. The datasets from BioGRID contained 9,698 nodes with 52,284 edges and 4,281 nodes with 7,415 edges (excluding pure high throughput experimental data) in human and mouse respectively. The HPRD dataset contained 9,453 nodes with 36,867 edges (excluding pure high throughput experimental data).

### Dataset of miRNA targets

Targets of miRNAs were downloaded from two different databases, which cover both human and mouse species. The first one is the miRecords dataset (http://mirecords.biolead.org/), which includes 284 miRNAs, 1,101 targets with 2,087 edges in the human and 145 miRNAs, 266 targets with 442 edges in the mouse [34]. The second database is TarBase (http://diana.cslab.ece.ntua.gr/DianaToolsNew/index.php), which includes 111 miRNAs, 862 targets with 1,093 edges in the human and 44 miRNAs, 75 targets with 104 edges in the mouse [35]. The targets of miRNAs listed in miRecords consist of experimentally verified targets and predicted targets which are an integration of predicted miRNA targets produced by 11 of the following miRNA target prediction tools: DIANA-microT, MicroInspector, miRanda, MirTarget2, miTarget, NBmiRTar, PicTar, PITA, RNA22, RNAhybrid, and TargetScan/TargertScanS. Since TarBase only includes experimentally verified miRNA targets, their scale of targets is less than those in miRecords. In order to obtain robust results, all the datasets used in this work were experimentally verified.

### Analysis of protein interaction network topological properties

Analyzing the topological properties of PPINs not only reveals the complex molecular interaction pathways, but also provides reference points for the detection of important transcriptional factors and downstream targets involved in maintaining the pluripotency of ESCs. Many topological properties of PPINs were used in this analysis. In this study, the Average Shortest Path Length (ASPL), Betweenness (BC), Closeness, Clustering Coefficient (CC), Degree, Eccentricity, Neighborhood Connectivity (NC), Radiality, Stress and Topological Coefficient (TC) were used to analyze the targets of OCT4, SOX2, NANOG and miRNAs (TarBases and miRecords) in the PPINs of the BioGRID and HPRD databases (Table 2). All the analyses were processed with Cytoscape and its NetworkAnalyzer tools.

**Table 2:** Definitions of the topological properties.

Property	Function	Description
Average Shortest Path Length (ASPL)	ASPL	The average number of steps for all shortest paths.
Betweenness Centrality (BC)	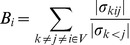	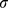 _k,j_ denotes shortest paths between node pairs K and j, 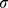 _kij_ denotes that pass through the node i.
Closeness Centrality (CC)	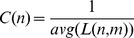	*L*(*n*,*m*) is the length of the shortest path between two nodes *n* and *m*. The Closeness centrality of each node is a number between 0 and 1.
Clustering Coefficient (CC)	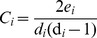	d_i_ is the number of neighbors of i, and e_i_ is the number of connected pairs between all neighbors of i.
Degree	d_i_	The number of links to node i.
Eccentricity	E	The maximum node eccentricity (E) can be desicribed as the network diameter, that is the largest distance between two nodes.
Neighborhood Connectivity (NC)	d	It is defined as the average connectivity of all neighbors of the node.
Radiality	R = D-ASPL+1	This attribute is a node centrality index computed by the diameter (D) of a node n's the connected component plus 1 and subtracting the average shortest path length (ASPL).
Stress	s	s of a node n is the number of shortest paths passing through n.
Topological Coefficient (TC)	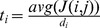	J(i, j) is the number of neighbors shared between the nodes i and j, plus one if there is a direct link between i and j. avg(J(i,j)) is the average value of J(i, j). d_i_ is degree of node i.

All the above topological properties can be used to measure the centrality of nodes in biological networks. General speaking, the higher the centrality of one node, the more important roles it plays in biological networks. For detailed description, we took some properties as examples to illustrate their meanings. ASPL is defined as the average shortest path length between a node and all the nodes in biological networks. Closeness centrality is defined as the reciprocal of the average shortest path length of one node which can be used as a measure of how fast information spreads from a given node to all other reachable nodes in biological networks. In undirected biological networks (such as PPINs), CC of a node is defined as the proportion of the observed connections between the neighbors of this node against the maximum number of possible connections among them. CC is used to indicate the close extent of the local neighborhood of one node. Degree is one simplest and most used topological index, which is defined as the number of nodes directly connected to a given node. TC is a relative measurement of the tendency of one node in biological networks to have shared interactive partners with other nodes. For more in-depth interpretation of these concepts, one can get the exact definitions of these topological properties from Table 2.

## Results

### The targets of “core” pluripotency TFs

A total of 2,512 human pluripotency “core” TF targets were identified in the collected articles. Among these targets, 42.9% were shared by at least two TFs, corresponding to 1,017 targets. The number of OCT4, SOX2 and NANOG targets was 623, 1,435 and 1,885 respectively, while the proportion shared by other TFs was 77.5%, 69.5% and 54.5% respectively (Figure 1A). Similar results were found in the mouse species. The total number of TF targets was 15,714, including 6,393 targets that were shared by two or three other TFs, accounting for 40.7% of the total number. Among the TF targets, 12,636 were OCT4 targets, 5,970 were SOX2 targets and 6,613 wee NANOG targets. Of these, 45.0%, 86.8% and 76.2%, respectively, were shared by other TFs (Figure 1B). As the results show, the target numbers and proportions that shared by different TFs were differences between human and mouse. The reason may be contributed from the research depth on human and mouse. As our manuscript show (Table 1), compared with the up to 8 literatures in mouse, there were only 2 literatures that contain at least two of the three “core” TF targets in human. As one extremely example, there was only 1 literature that contain OCT4 targets in human. In order to overcome the dataset bias and enrich the information in human, more works should be done for the genome wide target detection of these three “core” TFs in human.

### Mapping targets into PPINs

**Figure 2:**
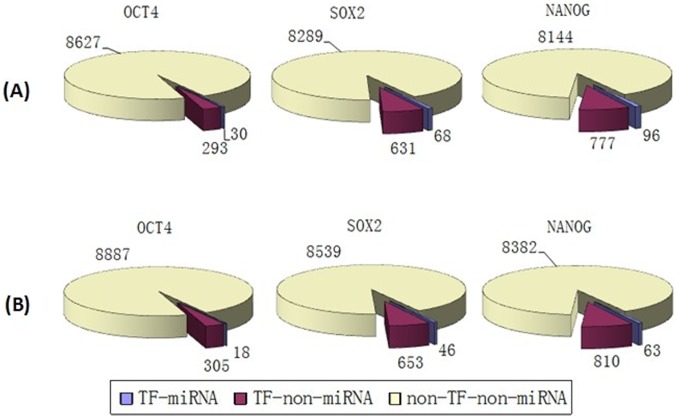
The distribution of targets of “core” pluripotency TFs in human BioGRID. (A) miRNA targets obtained from miRecords. (B) Targets of miRNAs from TarBase.

**Figure 3:**
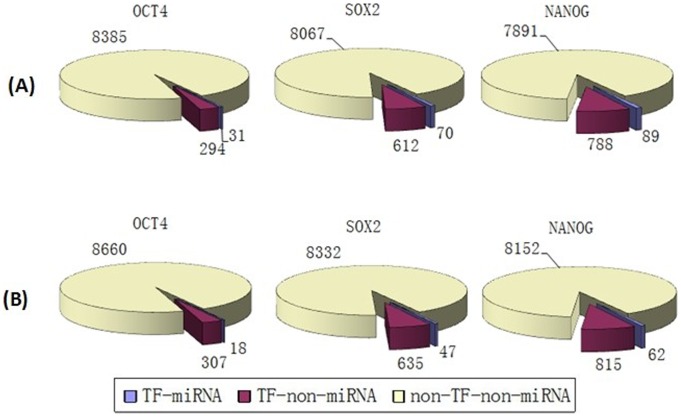
The distribution of targets of “core” pluripotency TFs in HPRD. (A) miRNA targets obtained from miRecords. (B) Targets of miRNAs from TarBase.

**Figure 4:**
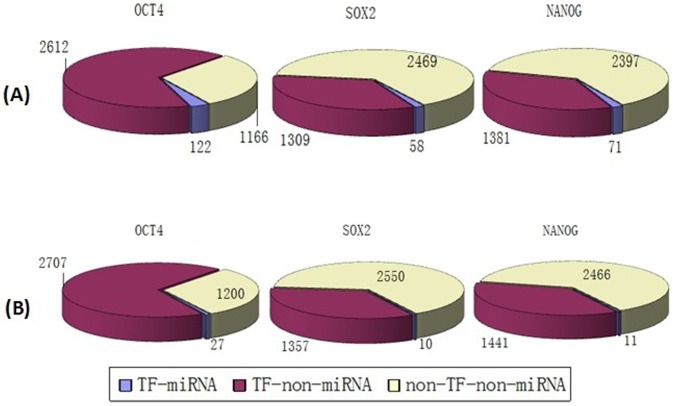
The distribution of targets of “core” pluripotency TFs in mouse BioGRID. (A) miRNA targets obtained from miRecords. (B) Targets of miRNAs from TarBase.

We mapped the targets of the three TFs and miRNAs into the PPINs and obtained sub-networks consisting only of “core” pluripotency TF targets and their direct neighbors (Figure S1 to S9). From both BioGRID and HPRD results, it was evident that the proportions of TF-miRNA targets (targets which are regulated by both these three “core” TFs and miRNAs) and TF-non-miRNA targets (targets which are regulated only by these three “core” TFs but not regulated by miRNAs) were smaller compared with the proportion of non-TF-non-miRNA genes (genes which are neither regulated by these three “core” TFs nor regulated by miRNAs) in both human and mouse (Figure 2, 3 and 4).

### Analysis of topological properties

The topological properties were analyzed with Cytoscape version 3.0.2, especially the NetworkAnalyzer tools. To begin with, the most connected components of protein-protein interaction networks were extracted from BioGRID, including 9,552 nodes with 52,202 edges in the human and 3,831 nodes with 7,123 edges in the mouse. The same analysis identified 9,205 nodes and 36,748 edges in the most connected component of the HPRD database. Finally, topological properties were analyzed with NetworkAnalyzer and filtered using strict statistical parameters (*P* values<0.05 with t test, processed with R version 3.0.2).

### Comparison of centrality properties between TF-targets and non-TF-targets

Following, we compared the topological properties between TF-targets (genes that are regulated by the “core” TFs, including OCT4, SOX2 and NANOG) and non-TF-targets (genes that are not regulated by any of the “core” TFs, including OCT4, SOX2 and NANOG). First we analyzed human PPINs. The results showed that the ASPL of SOX2-targets and NANOG-targets was shorter compared with non-targets, in both the BioGRID and HPRD datasets. We also found that radiality of SOX2-targets and NANOG-targets was greater than non-targets in both BioGRID and HPRD resources. Furthermore, the degree of the NANOG-targets was also significantly different compared with non-NANOG-targets in BioGRID and HPRD, indicating that many proteins are connected with NANOG-targets. Without consistent significant results, the SOX2-targets were only found to have higher degree values compared with non-SOX2-targets in HPRD, while similar results were not found in BioGRID. With regard to OCT4-targets, BC differed significantly between OCT4-targets and non-OCT4-targets in both BioGRID and HPRD databases, indicating that the shortest paths going through OCT4 targets were more than a random choice. This indicates that OCT4 targets may be internal module proteins and are more likely to locate in the hub position in networks. In summary, a certain degree of higher centrality in PPINs was found in human “core” pluripotency targets compared with non-TF-targets (Table 3).

**Table 3:** Human PPIN topological properties of TF-targets vs NON-TF-targets.

Property	Dataset	OCT4 vs NON-OCT4			SOX2 vs NON-SOX2			NANOG vs NON-NANOG		
		Average	Average	P	Average	Average	P	Average	Average	P
ASPL	BioGRID	3.574171	3.60704	0.2561	3.528778	3.611939	0.0001228	3.521464	3.614302	2.332E-6
	HPRD	4.093096	4.146839	0.1429	4.08806	4.149418	0.02393	4.097943	4.149802	0.04426
Betweenness	BioGRID	1.75347E-04	0.001484981	2.439E-04	6.74666E-04	0.001500916	0.1287	6.40797E-04	0.001520557	0.07553
	HPRD	2.79325E-04	0.0025712	1.69E-06	0.002882312	0.002462086	0.8179	0.002306237	0.002511442	0.8878
Closeness	BioGRID	0.2862341	0.290058	0.2253	0.2919629	0.2897727	0.424	2.92E-01	0.289702	0.288
	HPRD	0.2557632	0.2589428	0.5414	0.2573345	0.25895	0.6772	0.2589372	0.2588228	0.9757
CC	BioGRID	0.2101071	0.1828742	0.08325	0.1815801	0.1839522	0.8099	0.1899667	0.1831693	0.448
	HPRD	0.1045342	0.1030916	0.8997	0.09825781	0.10352095	0.4949	0.09893491	0.10357138	0.5041
Degree	BioGRID	18.32508	20.27829	0.316	29.69814	19.4765	0.1185	31.10767	19.13552	0.03128
	HPRD	7.855385	7.802695	0.9331	9.27566	7.690115	0.009551	9.070696	7.675023	0.008655
Eccentricity	BioGRID	8.198142	8.19328	0.9039	8.131617	8.198244	0.05012	8.136312	8.199093	3.91E-02
	HPRD	9.615385	9.573948	0.5599	9.612903	9.572455	0.4474	9.59293	9.573577	0.7064
NC	BioGRID	126.1499	119.2906	0.5021	122.932	119.254	0.5943	130.1599	118.4664	0.08831
	HPRD	39.22931	35.4478	0.05731	37.13338	35.45685	0.2265	36.60404	35.47286	0.3295
Radiality	BioGRID	0.7854858	0.7821837	0.1709	0.7889704	0.7817751	5.68E-05	0.7894008	0.7815906	1.308E-6
	HPRD	0.7784053	0.7740564	0.08891	0.7786679	0.773859	0.01069	0.7776415	0.7738546	0.0332
Stress	BioGRID	305873.1	450004.9	0.02379	934659.5	407185.9	0.2534	9.08E+05	399436.9	0.174
	HPRD	310804.1	357170	0.4041	447976.2	348391.2	0.1233	417604	349232.8	0.1987
TC	BioGRID	0.2021913	0.1865986	0.1279	0.1781781	0.1878123	0.1652	0.1821223	0.1876121	0.3843
	HPRD	0.2013462	0.1958896	0.6086	0.1893462	0.1966006	0.3103	0.1938195	0.1963081	0.7063

Similar results were also obtained in the mouse. For NANOG-targets, 6 measurements were found to differ significantly from those in non-NANOG-targets, including ASPL, Closeness, Degree, NC, Radiality and Stress. For SOX2-targets, 5 measurements in total were significantly different compared with non-SOX2-targets: ASPL, Closeness, Degree, NC and Radiality. For OCT4-targets, 5 measurements were found to differ from non-OCT4-targets, including BC, Degree, NC, Stress and TC. Taking these measurement results together, the target genes of “core” pluripotency transcription factors show higher centrality properties in mouse PPINs (Table 4).

**Table 4:** Mouse PPIN topological properties of TF-targets vs NON-TF-targets.

Property	OCT4 vs NON-OCT4			SOX2 vs NON-SOX2			NANOG vs NON-NANOG		
	Average	Average	P	Average	Average	P	Average	Average	P
ASPL	4.922332	4.918182	0.8876	4.874096	4.946149	0.01026	4.882961	4.943273	0.03216
BC	0.00119	0.000649	0.003523	0.0011367	0.0009639	0.4123	0.0013865	0.0008127	0.06287
Closeness	0.2084117	0.2085047	0.9332	0.2101989	0.2075007	0.01171	0.2100418	0.2075065	0.0171
CC	0.0942913	0.1020857	0.3649	0.0950919	0.0975313	0.7553	0.0964519	0.096816	0.9627
Degree	4.076054	2.910638	1.95E-06	4.196402	3.463356	0.01287	4.364989	3.341736	0.00176
Eccentricity	10.94014	10.8766	0.0646	10.9018	10.93072	0.3788	10.89157	10.9376	0.1595
NC	41.7789	55.00762	4.25E-06	41.12322	48.35415	0.003487	41.62068	48.29418	0.006982
Radiality	0.7548542	0.7551136	0.8876	0.757869	0.7533657	0.01026	0.7573149	0.7535454	0.03216
Stress	118484.92	65591.25	0.002851	117399.72	94174.79	0.263	141375.3	79456.66	0.03827
TC	0.1917032	0.1730731	0.02049	0.1852946	0.1863603	0.8874	0.1903151	0.1834669	0.3597

### Consistency analysis of multiple “core” pluripotency TF regulations

Through the analysis of the distributions of “core” pluripotency TF targets, we identified many genes regulated by at least two TFs (Figure 1). This result indicates that these TFs may be involved in complex interactions and execute similar functions synergistically as cells progress along the pathway of ESC development. To investigate this further, we continued to explore cooperation between the TF regulators through their topological properties. As expected, no difference in the centrality properties was found between 1TF-targtes, 2TF-targets and 3TF-targets in human PPINs, whichever protein-protein interaction datasets were used (Table 5).

**Table 5:** Human PPIN topological properties of 1TF (OCT4 or SOX2 or NANOG), 2TF (OCT4-SOX2 or OCT4-NANOG or SOX2-NANOG) and 3TF (OCT4-SOX2-NANOG) targets.

Property	Dataset	1TF vs 2TF			1TF vs 3TF			2TF vs 3TF		
		Average	Average	P	Average	Average	P	Average	Average	P
ASPL	BioGRID	3.525956	3.525934	0.9995	3.525956	3.554369	0.4811	3.525934	3.554369	0.5418
	HPRD	4.135929	4.073781	0.1973	4.135929	4.068019	0.205	4.073781	4.068019	0.9237
BC	BioGRID	0.0002838	0.0011167	0.2859	0.00028386	0.00019839	0.1722	0.00111673	0.00019839	0.2389
	HPRD	0.0034177	0.0024444	0.738	0.00341772	0.00031196	0.1544	0.00244448	0.00031196	0.2699
Closeness	BioGRID	0.2914932	0.2945455	0.5333	0.2914932	0.2859754	0.1332	0.2945455	0.2859754	0.08464
	HPRD	0.2549381	0.2613242	0.3607	0.2549381	0.2567622	0.8113	0.2613242	0.2567622	0.6061
CC	BioGRID	0.1903692	0.1801044	0.5289	0.1903692	0.2027773	0.5845	0.1801044	0.2027773	0.3524
	HPRD	0.1009959	0.0938428	0.5838	0.10099596	0.1054617	0.7844	0.09384283	0.1054617	0.5175
Degree	BioGRID	25.50715	38.22067	0.3236	25.50715	18.89674	0.0867	38.22067	18.89674	0.1302
	HPRD	8.235747	10.078035	0.08782	8.235747	8.314917	0.9376	10.078035	8.314917	0.1749
Eccentricity	BioGRID	8.135135	8.125698	0.876	8.135135	8.184783	0.344	8.125698	8.184783	0.3633
	HPRD	9.656394	9.586705	0.4633	9.656394	9.563536	0.3844	9.586705	9.563536	0.8494
NC	BioGRID	129.8866	117.0611	0.2598	129.8866	135.4983	0.7446	117.0611	135.4983	0.3063
	HPRD	37.48394	35.62533	0.3923	37.48394	39.03577	0.6091	35.62533	39.03577	0.2953
Radiality	BioGRID	0.7891725	0.7889236	0.9363	0.7891725	0.7871359	0.5433	0.7889236	0.7871359	0.6429
	HPRD	0.7753447	0.7795725	0.2062	0.7753447	0.7796719	0.2382	0.7795725	0.7796719	0.9807
Stress	BioGRID	4.84E+05	1460438	2.79E-01	484355	352091.7	0.2163	1460438	352091.7	0.2189
	HPRD	350199.8	518785.5	0.1401	350199.8	343444.9	0.941	518785.5	343444.9	0.1942
TC	BioGRID	1.83E-01	0.1775335	6.09E-01	0.1834824	0.1944252	0.469	0.1775335	0.1944252	0.3052
	HPRD	1.98E-01	0.1792437	1.33E-01	0.1979256	0.2063739	0.5757	0.1792437	0.2063739	0.105

Similar results were also found in the mouse. Among the ten centrality properties, none of them was found to differ between 1TF-targets and 2TF-targets. When compared with 3TF-targets, only ASPL, Closeness and Radiality were found to be different from those of 2TF-targets. The greatest diversity was found between the groups of the 1TF-targets and 3TF-targets, where we found differences in five measurements: ASPL, Closeness, Eccentricity, Radiality and Stress (Table 6).

**Table 6:** Mouse PPIN topological properties of 1TF (OCT4 or SOX2 or NANOG), 2TF (OCT4-SOX2 or OCT4-NANOG or SOX2-NANOG) and 3TF (OCT4-SOX2-NANOG) Targets.

Property	1TF vs 2TF			1TF vs 3TF			2TF vs 3TF		
	Average	Average	P	Average	Average	P	Average	Average	P
ASPL	4.952508	4.976565	0.552	4.952508	4.829478	0.0005945	4.976565	4.829478	0.000944
BC	0.0009476	0.0014171	0.4349	0.0009476	0.0012905	0.1084	0.0014171	0.0012905	0.8358
Closeness	0.2069969	0.2064969	0.7374	0.2069969	0.2120813	0.0002244	0.2064969	0.2120813	0.0008233
CC	0.0948301	0.093984	0.9363	0.0948301	0.0971206	0.8194	0.093984	0.0971206	0.792
Degree	3.787031	3.902511	0.822	3.787031	4.542964	0.06774	3.902511	4.542964	0.2474
Eccentricity	10.96587	10.96307	0.9528	10.96587	10.86924	0.02109	10.96307	10.86924	0.06803
NC	43.31362	41.09661	0.4983	43.31362	41.05602	0.4679	41.09661	41.05602	0.991
Radiality	0.7529682	0.7514647	0.552	0.7529682	0.7606576	0.0005945	0.7514647	0.7606576	0.000944
Stress	91310.31	140625.7	0.3889	91310.31	135029.88	0.04418	140625.7	135029.88	0.9252
TC	0.1945123	0.189662	0.6452	0.1945123	0.1872613	0.4493	0.189662	0.1872613	0.8355

### Modularity within the inner “core” pluripotency TFs with shortest path length analysis

When we investigated the regulation of multiply “core” pluripotency TFs during the development of ESCs in PPINs, one question was triggered about whether there are closer relationships between targets of these TFs. One hypothesis suggests that the connections within and between TF targets should be closer than between other genes in PPINs. In other words, module properties are expected within and between the “core” pluripotency TFs. In order to verify this hypothesis, we performed shortest path length (SPL) analysis across human and mouse species. We found that the background averages of PPIN SPLs were 4.227, 4.201 and 5.125 in HPRD, human BioGRID and mouse BioGRID respectively. First, the smaller SPLs were detected within TF targets compared with the background SPLs. As shown in Table 7, 8 and 9, the SPLs of TF targets, including OCT4, SOX2 and NANOG, were all smaller than those of other proteins in the PPINs, no matter which source of PPIN data was used. Second, the common targets of at least two TFs showed smaller SPLs compared with other proteins in the PPINs (Table 7, 8 and 9). When the number of TFs in combination was 2, the corresponding *P* values of the t tests were 4.99E-5, 0.001 and 3.90E-286; while when the number was 3, the *P* value was 7.98E-258 for values from the HPRD database (Table 7). Similar results were found for other databases (Table 8 and 9). Finally, we compared the distances between groups of different TF targets with SPLs and found that they were significantly different, especially when the group comprised the common targets of three TFs (Table 7, 8 and 9). From the HPRD and mouse BioGRID databases we found that the SPLs of three TFs were smaller than those of most other groups, but similar results were not observed in the PPIN of the human BioGRID. Summarizing the above results, we found module properties not only within the targets regulated by common or multiple “core” pluripotency TFs but also between the groups of targets regulated by different TFs.

**Table 7:** Analysis of shortest path length in HPRD.

	only-OCT4	only-SOX2	only-NANOG	OCT4-SOX2	OCT4-NANOG	SOX2-NANOG	OCT4-SOX2-NANOG
SPL	4.050	4.097	4.170	4.043	4.098	4.041	3.960
Others	1.95E-20	2.25E-72	1.93E-53	4.99E-05	0.001	3.90E-286	7.98E-258
only-SOX2	0.017						
only-NANOG	2.93E-09	1.91E-18					
OCT4-SOX2	0.882	0.221	0.007				
OCT4-NANOG	0.240	0.978	0.062	0.349			
SOX2-NANOG	0.627	7.05E-11	2.10E-88	0.961	0.116		
OCT4-SOX2-NANOG	1.63E-06	2.31E-43	1.45E-126	0.046	5.28E-05	1.08E-19	

(In this table, SPL indicates shortest path and the first line is the value of shortest path length of the targets; others is the p-value).

**Table 8:** Analysis of shortest path length in human BioGRID.

	only-OCT4	only-SOX2	only-NANOG	OCT4-SOX2	OCT4-NANOG	SOX2-NANOG	OCT4-SOX2-NANOG
SPL	4.221	3.865	3.966	3.862	4.557	3.944	3.989
Others	4.37E-01	2.00E-323	0.00E+00	2.59E-08	1.43E-17	0.00E+00	1.76E-111
only-SOX2	2.27E-46						
only-NANOG	7.13E-23	3.37E-26					
OCT4-SOX2	9.52E-09	0.958	0.080				
OCT4-NANOG	6.70E-10	7.71E-69	2.37E-46	2.51E-14			
SOX2-NANOG	4.46E-26	9.35E-15	3.45E-03	0.171191	1.24E-48		
OCT4-SOX2-NANOG	1.97E-22	2.57E-28	2.49E-02	0.01433	1.92E-51	2.24E-05	

(In this table, SPL indicates shortest path length and the first line is the value of shortest path length of the targets; others is the p-value).

**Table 9:** Analysis of shortest path length in mouse BioGRID.

	only-OCT4	only-SOX2	only-NANOG	OCT4-SOX2	OCT4-NANOG	SOX2-NANOG	OCT4-SOX2-NANOG
SPL	5.214	4.847	4.705	4.959	4.952	4.840	4.765
Others	0.00E+00	5.35E-89	0.00E+00	2.44E-131	2.93E-155	5.94E-75	0.00E+00
only-SOX2	1.21E-155						
only-NANOG	0.00E+00	8.31E-24					
OCT4-SOX2	3.17E-283	2.33E-11	5.17E-116				
OCT4-NANOG	0.00E+00	2.14E-10	2.44E-114	4.92E-01			
SOX2-NANOG	5.59E-129	7.10E-01	6.51E-17	3.34E-10	2.01E-09		
OCT4-SOX2-NANOG	0.00E+00	2.86E-08	1.35E-11	2.04E-139	2.40E-139	6.23E-06	

(In this table, SPL indicates shortest path length and the first line is the value of shortest path length of the targets; others is the p-value).

### Post-transcriptional regulation effects on “core” pluripotency TF targets

**Figure 5:**
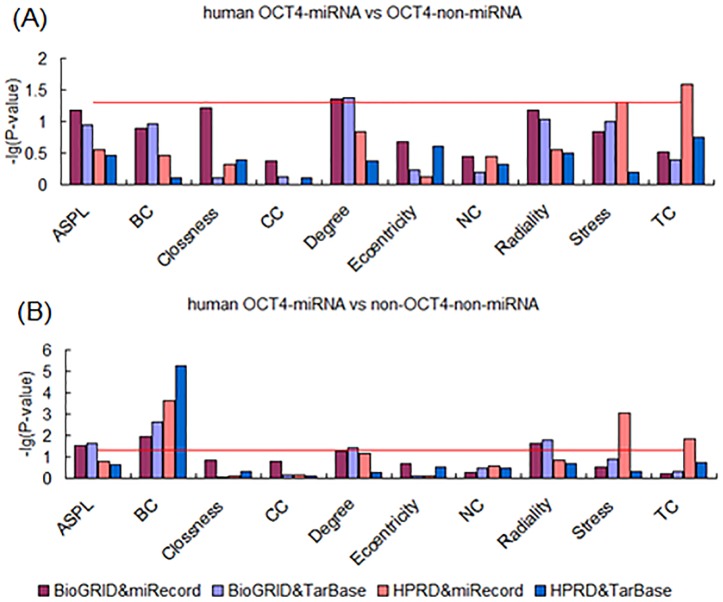
Analysis of topological properties of OCT4-miRNA-targets in human PPINs. (A) Comparison of OCT4-miRNA and OCT4-non-miRNA targets. (B) Comparison of OCT4-miRNA and non-OCT4-non-miRNA targets.

**Figure 6:**
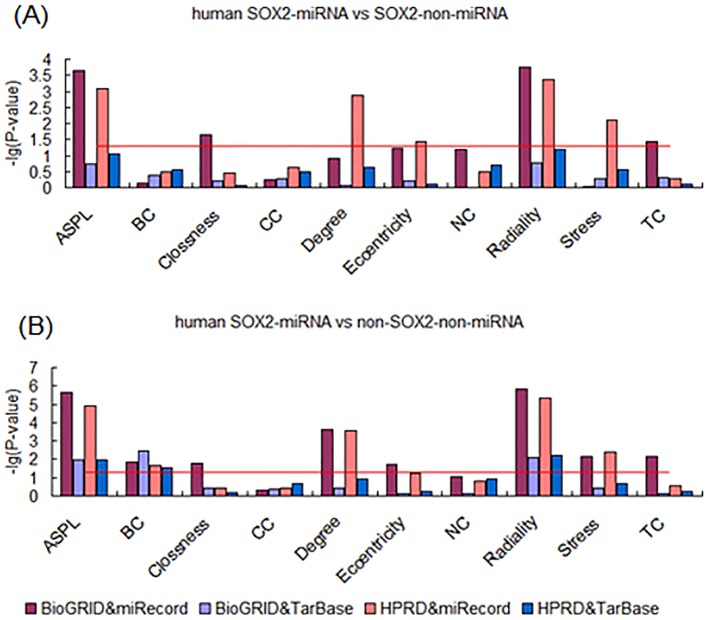
Analysis of topological properties of SOX2-miRNA-targets in human PPINs. (A) Comparison of SOX2-miRNA and SOX2-non-miRNA targets. (B) Comparison of SOX2-miRNA and non-SOX2-non-miRNA targets.

**Figure 7:**
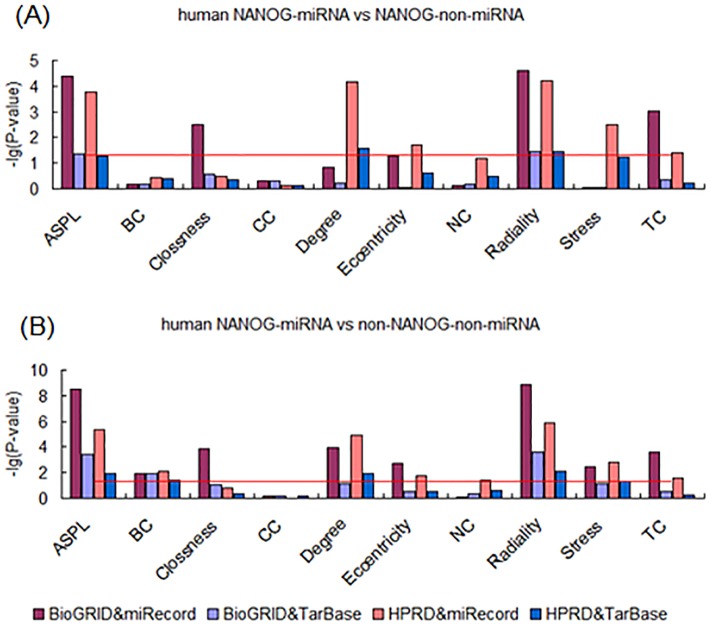
Analysis of topological properties of NANOG-miRNA-targets in human PPINs. (A) Comparison of NANOG-miRNA and NANOG-non-miRNA targets. (B) Comparison of NANOG-miRNA and non-NANOG-non-miRNA targets.

Based on the evidence that epigenetic regulation may play important roles in ESC development, we attempted to analyze the effects of post-transcriptional regulation on the targets of “core” pluripotency TFs in PPINs. As typical forms of post-transcriptional regulation, miRNA targets were obtained for further analysis from two different sources, including miRecords and TarBase. Results show that there were some significant different characteristics between TF-miRNA targets and TF-non-miRNA targets in the both two PPINs from human BioGRID and HPRD databases. The differences were found in both miRecords and TarBase. First, we found that OCT4-miRNA targets were different from OCT4-non-miRNA targets in several centrality properties, including Degree, Stress and TC (Figure 5). Second, observation of both SOX2-miRNA targets and NANOG-miRNA targets revealed that they differed from TF-non-miRNA targets in several measurements. The measurements that differed between SOX2-miRNA and SOX2-non-miRNA targets were ASPL, Closeness, Degree, Eccentricity, Radiality, Stress and TC (Figure 6). And the measurements, including ASPL, Clossness, Degree, Eccentricity, Radiality, Stress and TC, were different between NANOG-miRNA targets and NANOG-non-miRNA targets (Figure 7). Furthermore, these results were observed across both the BioGRID and HPRD protein-protein interaction databases and the miRNA target databases miRecords, TarBase.

Further, in order to identify the more obvious effects of miRNA regulation on the development of ESCs, we compared TF-miRNA target genes to non-TF-non-miRNA target genes in the human. Interestingly, as expected we found a significant difference between TF-miRNA and non-TF-non-miRNA targets on the topological properties of components of PPINs. First, OCT4-miRNA targets were found to have higher centrality properties compared with non-OCT4-non-miRNA genes in PPINs, with higher values of ASPL, BC, Degree, Stress, Radiality and TC (Figure 5). Second, the measurements which were higher in SOX2-miRNA targets compared with non-SOX2-non-miRNA genes were ASPL, BC, Closeness, Degree, Eccentricity, Radiality, Stress and TC (Figure 6). Third, up to 9 measurements differed between NANOG-miRNA targets and non-NANOG-non-miRNA genes in PPINs, excluding CC (Figure 7). These results were supported by all the different data sources, including BioGRID, HPRD, miRecords and TarBase.

**Figure 8:**
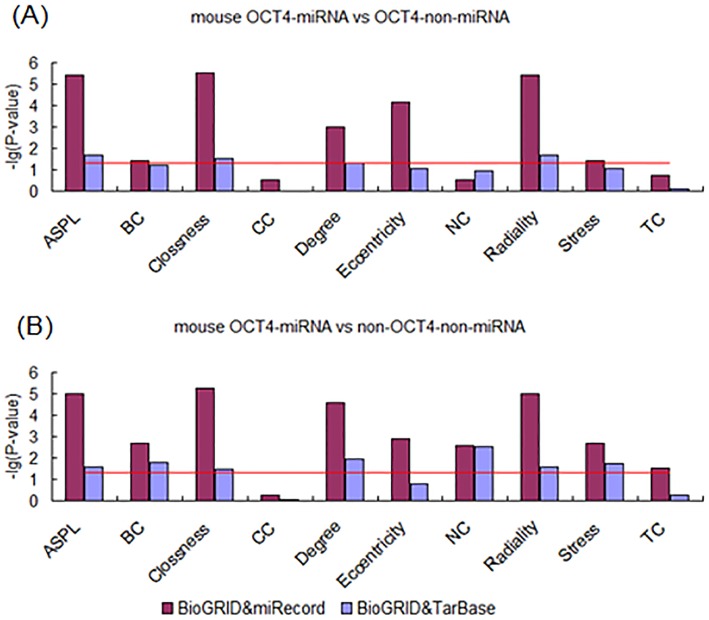
Analysis of topological properties of OCT4-miRNA-targets in mouse PPIN. (A) Comparison of OCT4-miRNA and OCT4-non-miRNA targets. (B) Comparison of OCT4-miRNA and non-OCT4-non-miRNA targets.

**Figure 9:**
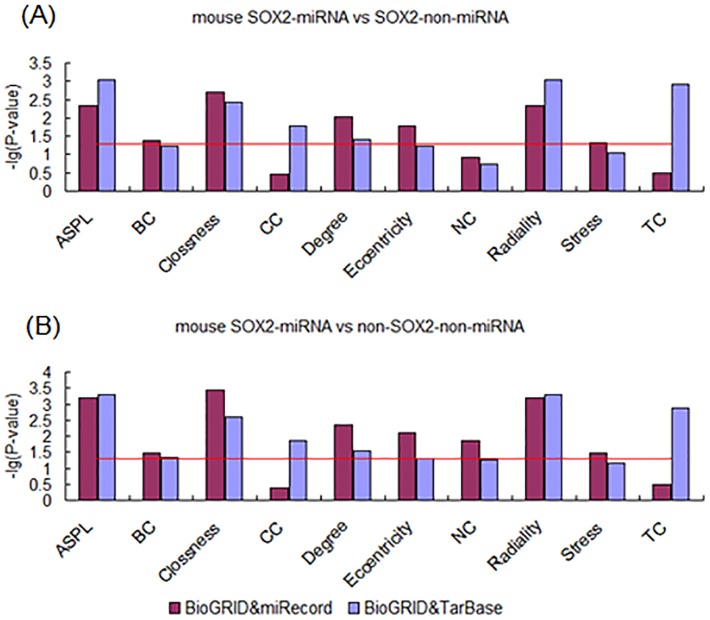
Analysis of topological properties of SOX2-miRNA-targets in mouse PPIN. (A) Comparison of SOX2-miRNA and SOX2-non-miRNA targets. (B) Comparison of SOX2-miRNA and non-SOX2-non-miRNA targets.

**Figure 10:**
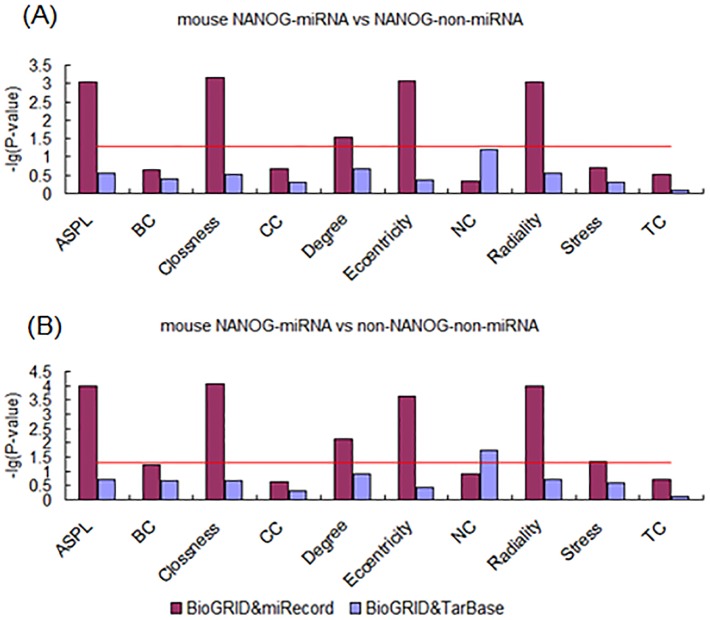
Analysis of topological properties of NANOG-miRNA-targets in mouse PPIN. (A) Comparison of NANOG-miRNA and NANOG-non-miRNA targets. (B) Comparison of NANOG-miRNA and non-NANOG-non-miRNA targets.

In order to overcome species bias, we conducted the same experiments in mouse ESCs. Interestingly, the results showed the same tendency as above in data from all the dataset sources. Firstly, the TF-miRNA targets also showed higher centrality properties compared with TF-non-miRNA targets (Figure 8, 9 and 10). The numbers of measurements that were different between TF-miRNA targets and TF-non-miRNA targets were up to 7, 9 and 5 in OCT4, SOX2 and NANOG respectively. The centrality properties that were not found to differ between OCT4-miRNA and OCT4-non-miRNA targets were CC, NC and TC. For SOX2, only NC showed no difference. The higher measurements in NANOG-miRNA targets included ASPL, Closeness, Degree, Eccentricity and Radiality. Second, the TF-miRNA targets differed from the non-TF-non-miRNA target genes in PPINs (Figure 8, 9 and 10). The numbers of measurements that were different between TF-miRNA targets and non-TF-non-miRNA targets were up to 9, 10 and 7 in OCT4, SOX2 and NANOG respectively. The measurements that were not found to differ were CC and BC, CC and TC in OCT4 and NANOG respectively. In summary, miRNAs play important roles during the development of ESCs and participate in complex interactions with “core” pluripotency TFs from a biological system perspective.

## Discussion

In this study, we globally analyzed the topological properties of targets of the “core” pluripotency TFs in PPINs, including OCT4, SOX2 and NANOG. In addition, the post-transciptional effects of miRNAs on these TFs were also analyzed in both human and mouse. Up to ten topological properties were included in the analysis process, including Shortest Path Length, Betweeness, Closeness, Cluster Coefficient, Degree, Eccentricity, Neighborhood Connectivity, Radiality, Stress and Topological Coefficient. All the above analyses were processed in three protein-protein interaction datasets (HPRD, human BioGRID and mouse BioGRID), two miRNA target datasets (miRecords and TarBase) and two species (human and mouse). Though there were different dataset scales of the three “core” TF targets between different databases and species, the common characteristic of these three “core” TFs in biological networks were still observed in our research. The use of several data sources and measurements in this study ensures the robust nature of the results obtained. Besides, all the above analysis was based on ESCs datasets. Consider the similar property of “stemness” with ESCs in many other types of stem cells, especially induced pluripotent stem cells, we infer that these three “core” TFs will have similar roles and characteristics in biological networks. With the increasing of high throughput datasets about targets of the “core” pluripotency TFs in other types of stem cells, similar research should be processed and compared with current results.

We found significant differences in centrality properties between “core” pluripotency TF-targets and non-TF-targets in PPINs. These results were widespread in HPRD, human BioGRID and mouse BioGRID datasets. The numbers of centrality properties were 6 and 8 in human and mouse respectively. The former contained ASPL, BC, Degree, Eccentricity, Radiality and Stress, while the latter comprised ASPL, BC, Closeness, Degree, NC, Radiality, Stress and TC. Comparing the two results, we found that ASPL, BC, Degree, Radiality and Stress were robust and only CC among the 10 measurements could not be detected, which is used to judge the close link of node neighborhoods in biological networks. The reason why CC did not appear in the analysis was not clear. It may be because the targets of “core” pluripotency TFs perform their functions in relative isolation which may help to avoid harm to the complex environment *in vivo* during the development of ESCs [36], [37]. With the higher central properties, these results indicate that targets of these three “core” TFs play more important roles than random genes during the development of ESCs.

We found synergistic regulation of multiple “core” pluripotency TFs during the development of ESCs. As we hypothesized, no difference in topological properties was found between 1TF-targets, 2TF-targets and 3TF-targets in human PPINs, including HPRD and human BioGRID. The same tendency was also found in mouse PPINs from BioGRID. Based on these results, it can be seen that although the number of TFs regulating common target genes increases from 1 to 3, their centrality properties do not increase accordingly. This indicates that pluripotency related genes may be regulated by 1 or 2 or even 3 TFs, but the genes are no different from the biological system viewpoint. In other words, the number of TFs that regulate the common pluripotency genes is not important. This means that the seemingly unnecessary TFs may provide compensatory regulation in the molecular pathways of ESC development [38]. Such compensatory regulation will conversely enhance the status of the common targets in a biological process. Through the consistent regulation of these TFs, the maintenance of the pluripotency of ESCs is rendered more reliable. As an example, the synergistic regulation of histone deacetylase 1 (HDAC1) by OCT4, SOX2 and NANOG plays important roles not only in the development of ESCs but also in the growth of tumor cells [39]-[41].

The module properties within the inner “core” pluripotency TFs were detected through the analysis of shortest path length in PPINs. Through these experiments, we found that the shortest path length of targets regulated by common TFs were smaller than those of randomly selected background values. This indicates that the genes regulated by common “core” pluripotency TFs are in close communication in PPINs and that this will be helpful in synergistically and quickly maintaining the pluripotency of ESCs [42]. Next, the shortest path lengths of target genes regulated by multiple TFs were further analyzed. Results show that these genes also have smaller shortest path lengths between each other. Third, we took the analysis of the extent of contact between groups of different TF targets and found that they were different, especially when the group comprised the common targets regulated by three TFs. In summary, we identified module properties not only within the targets regulated by common or multiple “core” pluripotency TFs but also between the groups of targets regulated by different TFs. The genes within module may have close and quickly information flowing which will help them to implement the common function of maintaining pluripotency during the development of ESCs.

We also found that miRNAs play important roles in the regulation of “core” pluripotency TFs during the development of ESCs as a way of post-transcriptional regulation in PPINs. The difference of centrality properties observed between TF-miRNA targets and TF-non-miRNA targets was found in both human and mouse PPINs. Further, the differences in topological properties between TF-miRNA targets and non-TF-non-miRNA target genes were more obvious in PPINs. These different centrality properties show different close extent and different importance in computational biological network view. Considering the effects that miRNAs impose to the “core” TFs on these properties in PPINs, it is observed that there is synergistic regulation between themselves. The synergistic regulation of “core” pluripotency TFs and miRNAs will enhance the function of targets. In consideration of the drastic effects of TFs and the minor regulation of miRNAs, the synergism of these three “core” TFs and miRNAs may help to achieve their aims of regulation about pluripotency, like one machine which has many gear wheel of different size. As an example, myocyte enhancer factor2 (MEF2) which is a target of both NANOG and miRNA, is an important transcription factor regulating the survival and development of many types of cells [43], [44]. One of its prominent functions is the control of gene transcription in cell differentiation. All of the other genes that regulated by NANOG and miRNAs (including Mxi1, Arid3b, Kit, Hoxa11, Hoxa7, Mef2c, Gja1, Myc, Hdac4 and Tmsb4x) are known to be related with stem cells. To further verify our results, we performed analysis of function categories with these ten targets in PIR and found that they are development proteins and related with transcription regulation (Table 10). Our results clearly reveal the effects of epigenetic regulations on the development of ESCs in biological networks, findings which are consistent with previous studies performed using molecular and cell technology [25], [45]. This finding may provide candidate pathway for deep detection about ESCs molecular mechanism from post transcriptional level.

**Table 10:** Function categories analysis about targets of NANOG and miRNAs in PIR.

Term	P Value
Transcription regulation	8.59E-05
Transcription	9.74E-05
Dna-binding	7.75E-04
Proto-oncogene	0.004849
Nucleus	0.005432
DNA binding	0.010331
Developmental protein	0.048812

## Supporting Information

Figure S1 **Sub-network of OCT4 in HPRD.**
(PDF)

Figure S2 **Sub-network of SOX2 in HPRD.**
(PDF)

Figure S3 **Sub-network of NANOG in HPRD.**
(PDF)

Figure S4 **Sub-network of OCT4 in human BioGRID.**
(PDF)

Figure S5 **Sub-network of SOX2 in human BioGRID.**
(PDF)

Figure S6 **Sub-network of NANOG in human BioGRID.**
(PDF)

Figure S7 **Sub-network of OCT4 in mouse BioGRID.**
(PDF)

Figure S8 **Sub-network of SOX2 in mouse BioGRID.**
(PDF)

Figure S9 **Sub-network of NANOG in mouse BioGRID.**
(PDF)

Table S1 **Targets of “core” pluripotency TFs in the human.**
(XLS)

Table S2 **Targets of “core” pluripotency TFs in the mouse.**
(XLS)

## References

[1] Thomson JA, Itskovitz-Eldor J, Shapiro SS, Waknitz MA, Swiergiel JJ, et al (1998) Embryonic stem cell lines derived from human blastocysts. Science 282: 1145–1147. 9804556 10.1126/science.282.5391.1145

[2] Martin GR (1981) Isolation of a pluripotent cell line from early mouse embryos cultured in medium conditioned by teratocarcinoma stem cells. Proceedings of the National Academy of Sciences 78: 7634–7638. 10.1073/pnas.78.12.7634 PMC349323 6950406

[3] Cowan CA, Atienza J, Melton DA, Eggan K (2005) Nuclear reprogramming of somatic cells after fusion with human embryonic stem cells. Science 309: 1369–1373. 16123299 10.1126/science.1116447

[4] Silva J, Chambers I, Pollard S, Smith A (2006) Nanog promotes transfer of pluripotency after cell fusion. Nature 441: 997–1001. 16791199 10.1038/nature04914

[5] Takahashi K, Yamanaka S (2006) Induction of pluripotent stem cells from mouse embryonic and adult fibroblast cultures by defined factors. Cell 126: 663–676. 16904174 10.1016/j.cell.2006.07.024

[6] Takahashi K, Tanabe K, Ohnuki M, Narita M, Ichisaka T, et al (2007) Induction of pluripotent stem cells from adult human fibroblasts by defined factors. Cell 131: 861–872. 18035408 10.1016/j.cell.2007.11.019

[7] Yu J, Vodyanik MA, Smuga-Otto K, Antosiewicz-Bourget J, Frane JL, et al (2007) Induced pluripotent stem cell lines derived from human somatic cells. Science 318: 1917–1920. 18029452 10.1126/science.1151526

[8] Maherali N, Sridharan R, Xie W, Utikal J, Eminli S, et al (2007) Directly reprogrammed fibroblasts show global epigenetic remodeling and widespread tissue contribution. Cell stem cell 1: 55–70. 18371336 10.1016/j.stem.2007.05.014

[9] Okita K, Ichisaka T, Yamanaka S (2007) Generation of germline-competent induced pluripotent stem cells. Nature 448: 313–317. 17554338 10.1038/nature05934

[10] Hou P, Li Y, Zhang X, Liu C, Guan J, et al (2013) Pluripotent stem cells induced from mouse somatic cells by small-molecule compounds. Science 341: 651–654. 23868920 10.1126/science.1239278

[11] Ahmad A, Li Y, Bao B, Kong D, Sarkar FH (2014) Epigenetic regulation of miRNA-cancer stem cells nexus by nutraceuticals. Molecular nutrition & food research 58: 79–86. 24272883 10.1002/mnfr.201300528 PMC3997058

[12] Kreso A, Dick JE (2014) Evolution of the Cancer Stem Cell Model. Cell stem cell 14: 275–291. 24607403 10.1016/j.stem.2014.02.006

[13] Liu S, Yin F, Zhang J, Wicha MS, Chang AE, et al. (2014) Regulatory Roles of miRNA in the Human Neural Stem Cell Transformation to Glioma Stem Cells. J Cell Biochem. 10.1002/jcb.24786 24519663

[14] Orkin SH (2005) Chipping away at the embryonic stem cell network. Cell 122: 828–830. 16179251 10.1016/j.cell.2005.09.002

[15] Nichols J, Zevnik B, Anastassiadis K, Niwa H, Klewe-Nebenius D, et al (1998) Formation of pluripotent stem cells in the mammalian embryo depends on the POU transcription factor Oct4. Cell 95: 379–391. 9814708 10.1016/s0092-8674(00)81769-9

[16] Niwa H (2007) How is pluripotency determined and maintained? Development 134: 635–646. 17215298 10.1242/dev.02787

[17] Avilion AA, Nicolis SK, Pevny LH, Perez L, Vivian N, et al (2003) Multipotent cell lineages in early mouse development depend on SOX2 function. Genes Dev 17: 126–140. 12514105 10.1101/gad.224503 PMC195970

[18] Chambers I, Colby D, Robertson M, Nichols J, Lee S, et al (2003) Functional expression cloning of Nanog, a pluripotency sustaining factor in embryonic stem cells. Cell 113: 643–655. 12787505 10.1016/s0092-8674(03)00392-1

[19] Mitsui K, Tokuzawa Y, Itoh H, Segawa K, Murakami M, et al (2003) The homeoprotein Nanog is required for maintenance of pluripotency in mouse epiblast and ES cells. Cell 113: 631–642. 12787504 10.1016/s0092-8674(03)00393-3

[20] Boyer LA, Lee TI, Cole MF, Johnstone SE, Levine SS, et al (2005) Core transcriptional regulatory circuitry in human embryonic stem cells. Cell 122: 947–956. 16153702 10.1016/j.cell.2005.08.020 PMC3006442

[21] Kim J, Chu J, Shen X, Wang J, Orkin SH (2008) An extended transcriptional network for pluripotency of embryonic stem cells. Cell 132: 1049–1061. 18358816 10.1016/j.cell.2008.02.039 PMC3837340

[22] Ng HH, Surani MA (2011) The transcriptional and signalling networks of pluripotency. Nat Cell Biol 13: 490–496. 21540844 10.1038/ncb0511-490

[23] Cassar PA, Stanford WL (2012) Integrating post-transcriptional regulation into the embryonic stem cell gene regulatory network. J Cell Physiol 227: 439–449. 21503874 10.1002/jcp.22787

[24] Kim J, Orkin SH (2011) Embryonic stem cell-specific signatures in cancer: insights into genomic regulatory networks and implications for medicine. Genome Med 3: 75–75. 22126538 10.1186/gm291 PMC3308030

[25] Marson A, Levine SS, Cole MF, Frampton GM, Brambrink T, et al (2008) Connecting microRNA genes to the core transcriptional regulatory circuitry of embryonic stem cells. Cell 134: 521–533. 18692474 10.1016/j.cell.2008.07.020 PMC2586071

[26] Anokye-Danso F, Trivedi CM, Juhr D, Gupta M, Cui Z, et al (2011) Highly efficient miRNA-mediated reprogramming of mouse and human somatic cells to pluripotency. Cell stem cell 8: 376–388. 21474102 10.1016/j.stem.2011.03.001 PMC3090650

[27] Melton C, Judson RL, Blelloch R (2010) Opposing microRNA families regulate self-renewal in mouse embryonic stem cells. Nature 463: 621–626. 20054295 10.1038/nature08725 PMC2894702

[28] Bartel DP (2004) MicroRNAs: genomics, biogenesis, mechanism, and function. Cell 116: 281–297. 14744438 10.1016/s0092-8674(04)00045-5

[29] Chatr-aryamontri A, Breitkreutz BJ, Heinicke S, Boucher L, Winter A, et al (2013) The BioGRID interaction database: 2013 update. Nucleic Acids Res 41: D816–D823. 23203989 10.1093/nar/gks1158 PMC3531226

[30] Prasad TK, Goel R, Kandasamy K, Keerthikumar S, Kumar S, et al (2009) Human protein reference database—2009 update. Nucleic Acids Res 37: D767–D772. 18988627 10.1093/nar/gkn892 PMC2686490

[31] Smoot ME, Ono K, Ruscheinski J, Wang PL, Ideker T (2011) Cytoscape 2.8: new features for data integration and network visualization. Bioinformatics 27: 431–432. 21149340 10.1093/bioinformatics/btq675 PMC3031041

[32] Assenov Y, Ramírez F, Schelhorn SE, Lengauer T, Albrecht M (2008) Computing topological parameters of biological networks. Bioinformatics 24: 282–284. 18006545 10.1093/bioinformatics/btm554

[33] Doncheva NT, Assenov Y, Domingues FS, Albrecht M (2012) Topological analysis and interactive visualization of biological networks and protein structures. Nat Protoc 7: 670–685. 22422314 10.1038/nprot.2012.004

[34] Xiao F, Zuo Z, Cai G, Kang S, Gao X, et al (2009) miRecords: an integrated resource for microRNA–target interactions. Nucleic Acids Res 37: D105–D110. 18996891 10.1093/nar/gkn851 PMC2686554

[35] Vergoulis T, Vlachos IS, Alexiou P, Georgakilas G, Maragkakis M, et al (2012) TarBase 6.0: capturing the exponential growth of miRNA targets with experimental support. Nucleic Acids Res 40: D222–D229. 22135297 10.1093/nar/gkr1161 PMC3245116

[36] Barabasi AL, Oltvai ZN (2004) Network biology: understanding the cell's functional organization. Nature Reviews Genetics 5: 101–113. 10.1038/nrg1272 14735121

[37] Zhang P, Wang J, Li X, Li M, Di Z, et al (2008) Clustering coefficient and community structure of bipartite networks. Physica A: Statistical Mechanics and its Applications 387: 6869–6875.

[38] Archer TC, Jin J, Casey ES (2011) Interaction of Sox1, Sox2, Sox3 and Oct4 during primary neurogenesis. Dev Biol 350: 429–440. 21147085 10.1016/j.ydbio.2010.12.013 PMC3033231

[39] Reichmann J, Crichton JH, Madej MJ, Taggart M, Gautier P, et al (2012) Microarray analysis of LTR retrotransposon silencing identifies Hdac1 as a regulator of retrotransposon expression in mouse embryonic stem cells. PLoS Comput Biol 8: e1002486. 22570599 10.1371/journal.pcbi.1002486 PMC3343110

[40] Liang J, Wan M, Zhang Y, Gu P, Xin H, et al (2008) Nanog and Oct4 associate with unique transcriptional repression complexes in embryonic stem cells. Nat Cell Biol 10: 731–739. 18454139 10.1038/ncb1736

[41] Pan L, Lu J, Huang B (2007) HDAC inhibitors: a potential new category of anti-tumor agents. Cell Mol Immunol 4: 337–343. 17976313

[42] Wong DJ, Liu H, Ridky TW, Cassarino D, Segal E, et al (2008) Module map of stem cell genes guides creation of epithelial cancer stem cells. Cell stem cell 2: 333–344. 18397753 10.1016/j.stem.2008.02.009 PMC2628721

[43] Fazi F, Nervi C (2008) MicroRNA: basic mechanisms and transcriptional regulatory networks for cell fate determination. Cardiovasc Res 79: 553–561. 18539629 10.1093/cvr/cvn151

[44] Wilson KD, Hu S, Venkatasubrahmanyam S, Fu JD, Sun N, et al (2010) Dynamic microrna expression programs during cardiac differentiation of human embryonic stem cells role for mir-499. Circ Cardiovasc Genet 3: 426–435. 20733065 10.1161/CIRCGENETICS.109.934281 PMC3057038

[45] Ivey KN, Muth A, Arnold J, King FW, Yeh RF, et al (2008) MicroRNA regulation of cell lineages in mouse and human embryonic stem cells. Cell stem cell 2: 219–229. 18371447 10.1016/j.stem.2008.01.016 PMC2293325

[46] Ben-Porath I, Thomson MW, Carey VJ, Ge R, Bell GW, et al (2008) An embryonic stem cell–like gene expression signature in poorly differentiated aggressive human tumors. Nat Genet 40: 499–507. 18443585 10.1038/ng.127 PMC2912221

[47] Loh YH, Wu Q, Chew JL, Vega VB, Zhang W, et al (2006) The Oct4 and Nanog transcription network regulates pluripotency in mouse embryonic stem cells. Nat Genet 38: 431–440. 16518401 10.1038/ng1760

[48] Chen X, Xu H, Yuan P, Fang F, Huss M, et al (2008) Integration of external signaling pathways with the core transcriptional network in embryonic stem cells. Cell 133: 1106–1117. 18555785 10.1016/j.cell.2008.04.043

[49] Cole MF, Johnstone SE, Newman JJ, Kagey MH, Young RA (2008) Tcf3 is an integral component of the core regulatory circuitry of embryonic stem cells. Genes Dev 22: 746–755. 18347094 10.1101/gad.1642408 PMC2275428

[50] Pardo M, Lang B, Yu L, Prosser H, Bradley A, et al (2010) An expanded Oct4 interaction network: implications for stem cell biology, development, and disease. Cell stem cell 6: 382–395. 20362542 10.1016/j.stem.2010.03.004 PMC2860244

[51] Ang YS, Tsai SY, Lee DF, Monk J, Su J, et al (2011) Wdr5 mediates self-renewal and reprogramming via the embryonic stem cell core transcriptional network. Cell 145: 183–197. 21477851 10.1016/j.cell.2011.03.003 PMC3097468

[52] Whyte WA, Orlando DA, Hnisz D, Abraham BJ, Lin CY, et al (2013) Master transcription factors and mediator establish super-enhancers at key cell identity genes. Cell 153: 307–319. 23582322 10.1016/j.cell.2013.03.035 PMC3653129

