# Supplementary figures and images for: Synergistic Transcriptional and Post-Transcriptional Regulation of ESC Characteristics by Core Pluripotency Transcription Factors in Protein-Protein Interaction Networks
Source: PLoS One. 2014 Aug 29;9(8):e105180. doi: 10.1371/journal.pone.0105180 (PMC4149371; doi:10.1371/journal.pone.0105180)

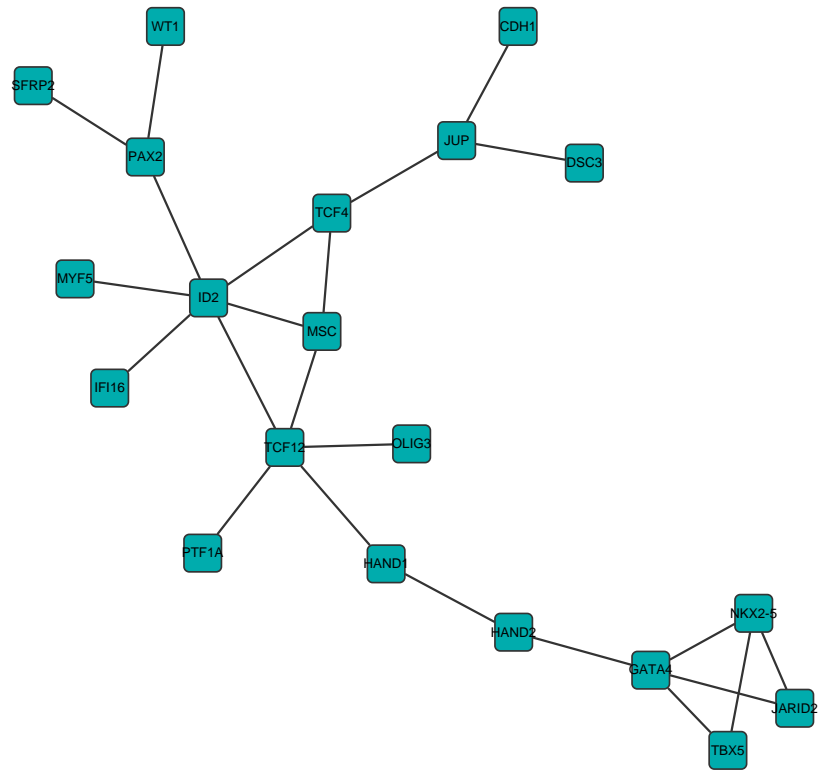

Supplement: Figure S1 — Sub-network of OCT4 in HPRD. (PDF) [file pone.0105180.s001.pdf]

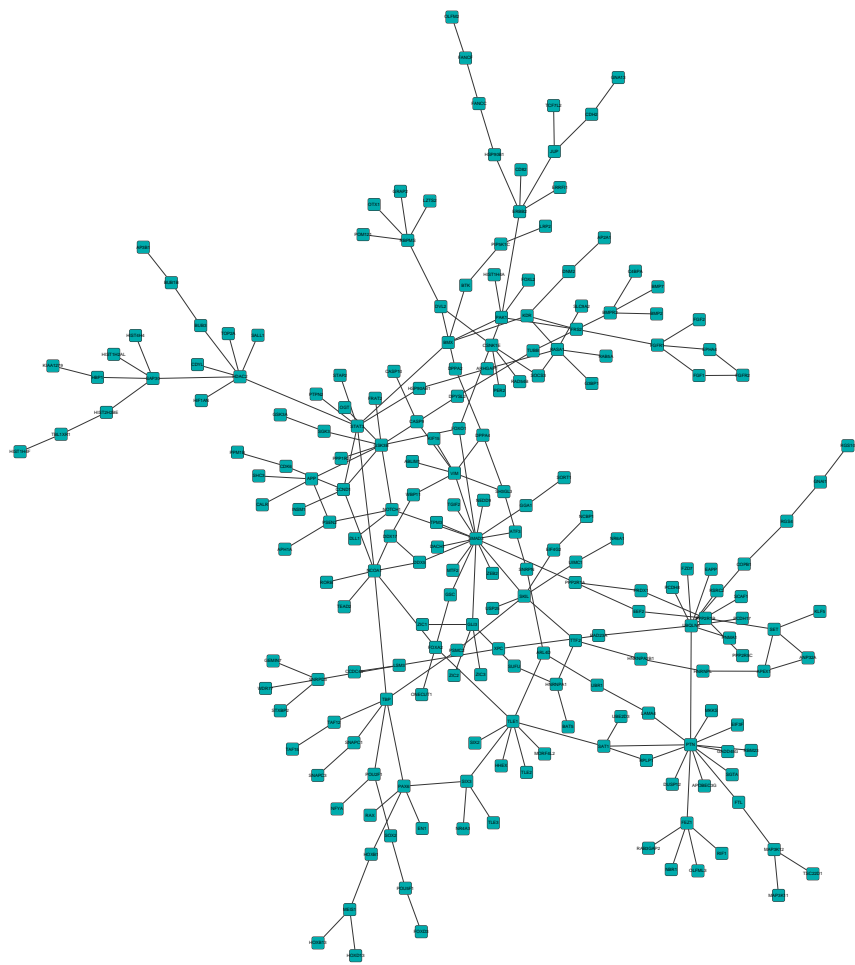

Supplement: Figure S2 — Sub-network of SOX2 in HPRD. (PDF) [file pone.0105180.s002.pdf]

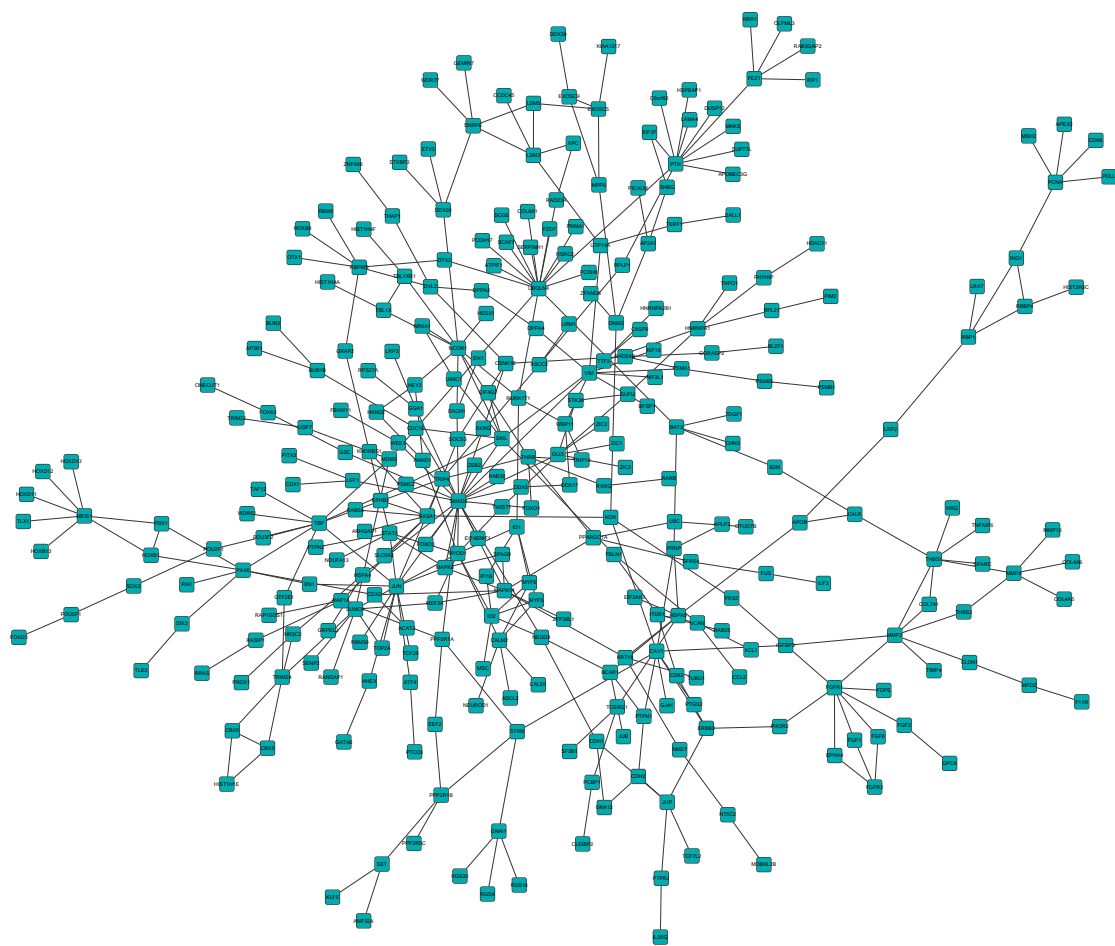

Supplement: Figure S3 — Sub-network of NANOG in HPRD. (PDF) [file pone.0105180.s003.pdf]

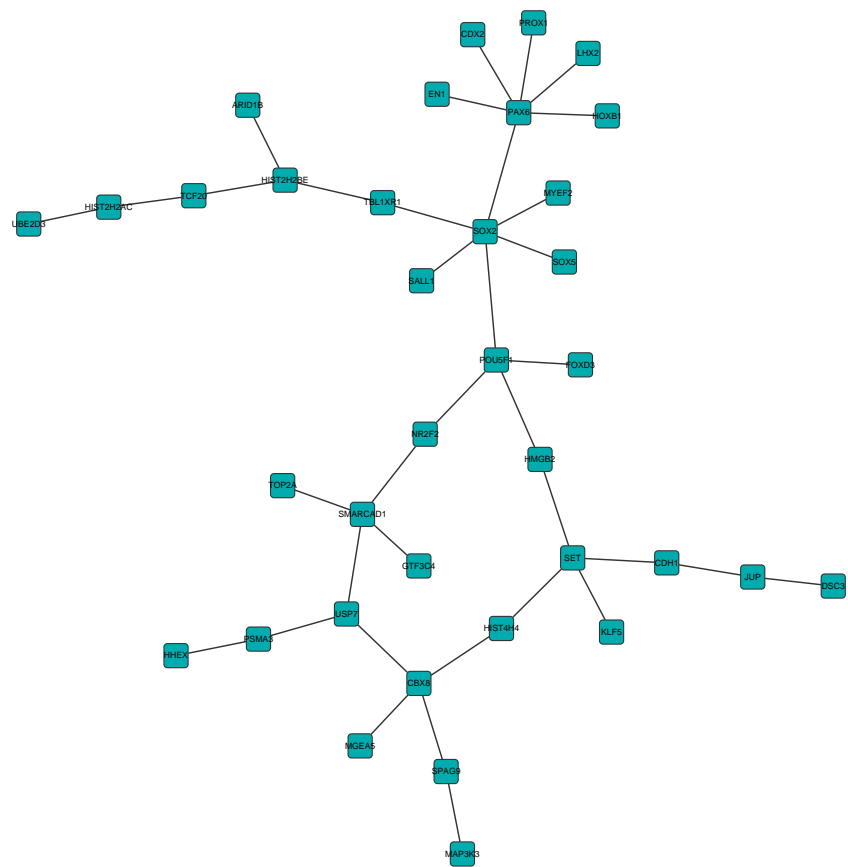

Supplement: Figure S4 — Sub-network of OCT4 in human BioGRID. (PDF) [file pone.0105180.s004.pdf]

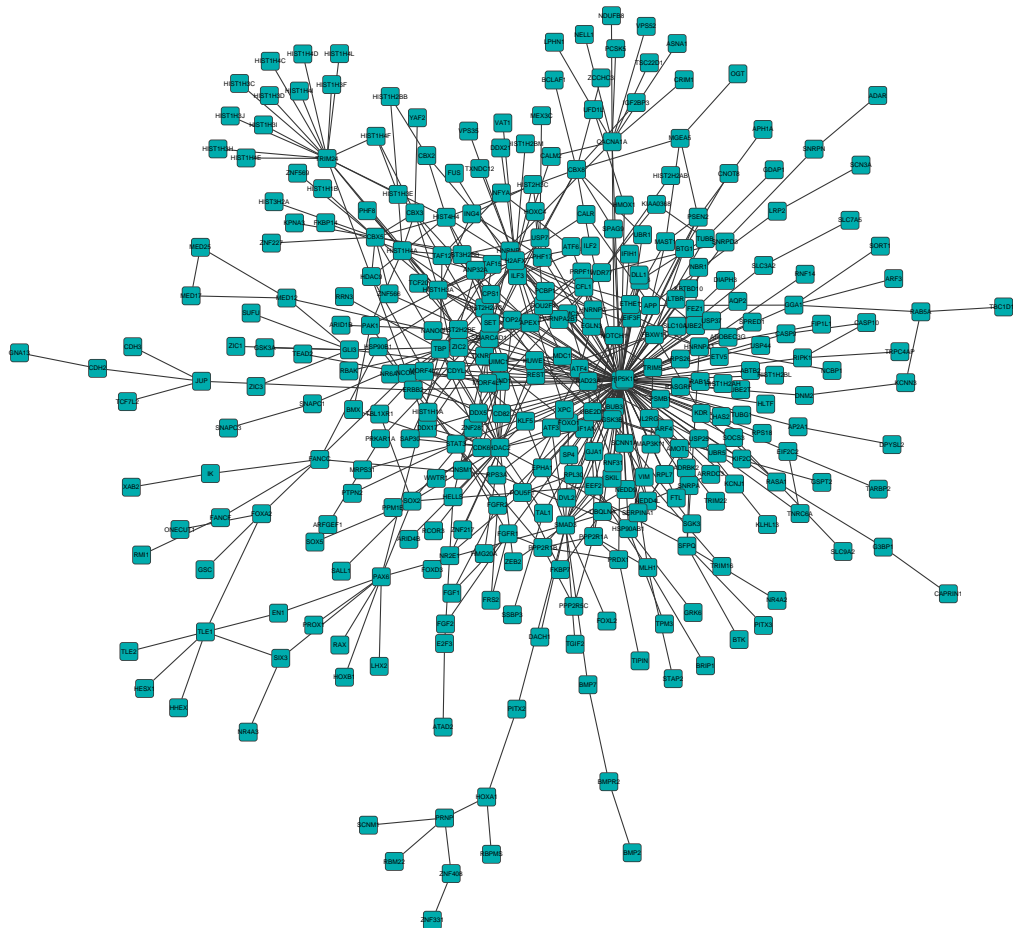

Supplement: Figure S5 — Sub-network of SOX2 in human BioGRID. (PDF) [file pone.0105180.s005.pdf]

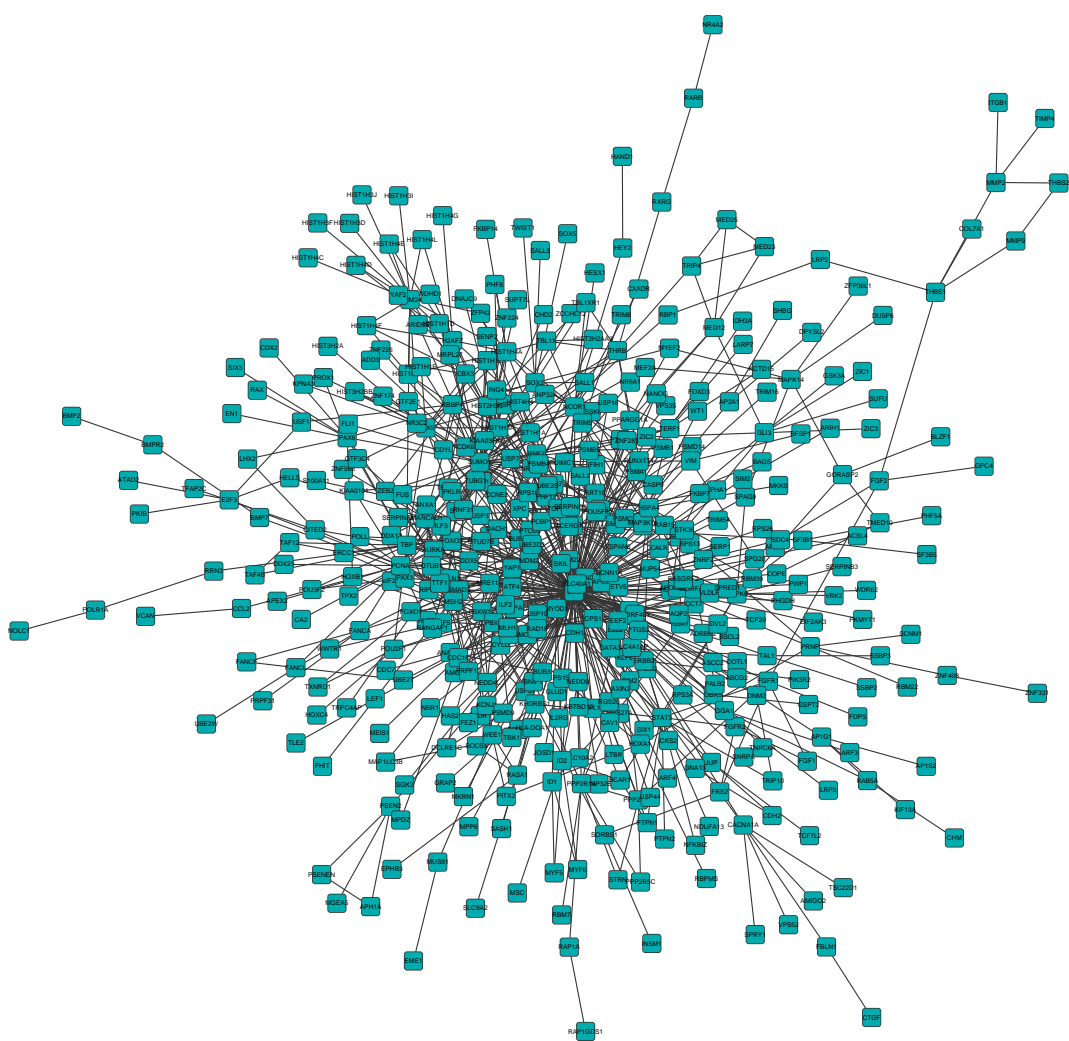

Supplement: Figure S6 — Sub-network of NANOG in human BioGRID. (PDF) [file pone.0105180.s006.pdf]

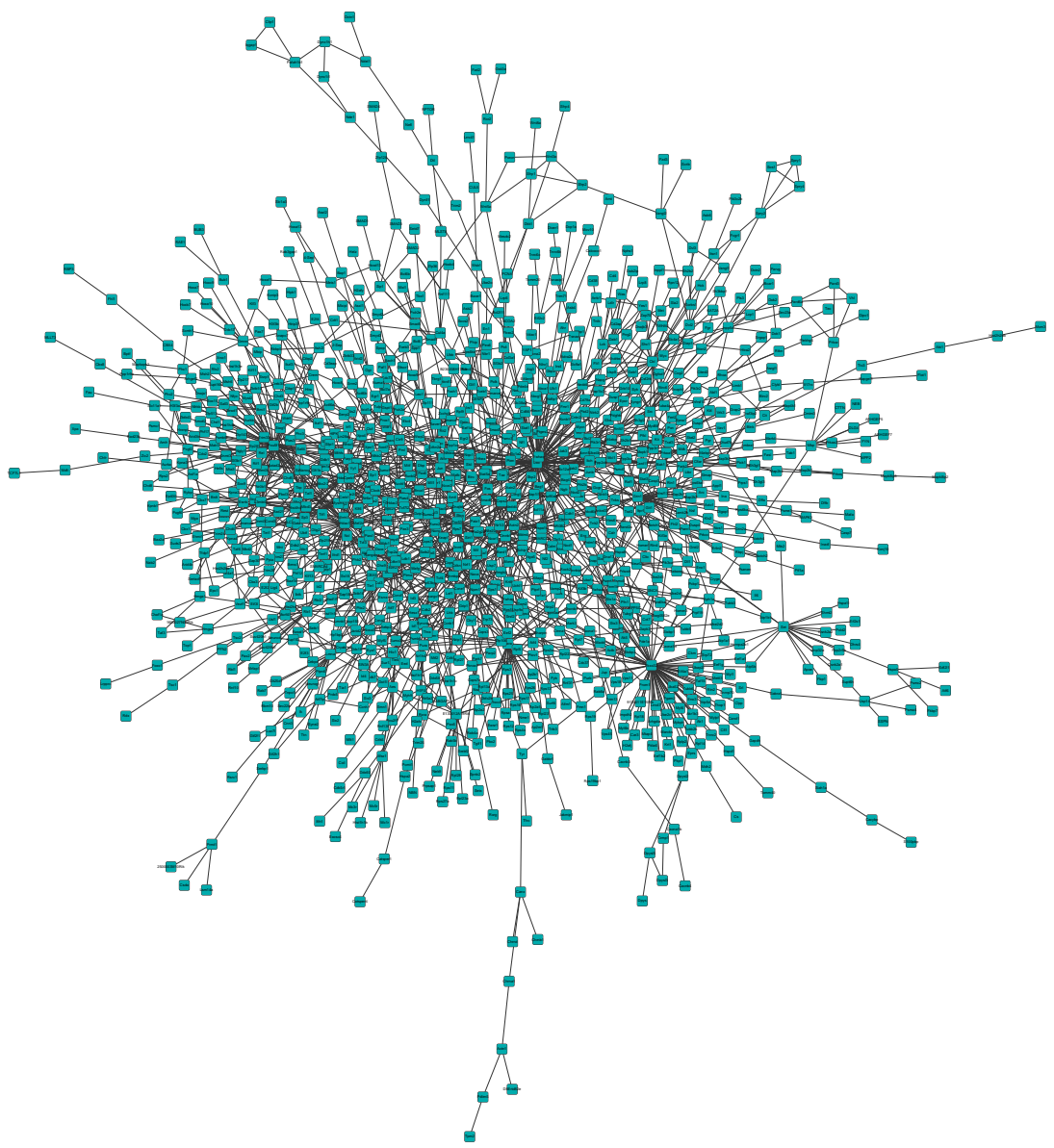

Supplement: Figure S7 — Sub-network of OCT4 in mouse BioGRID. (PDF) [file pone.0105180.s007.pdf]

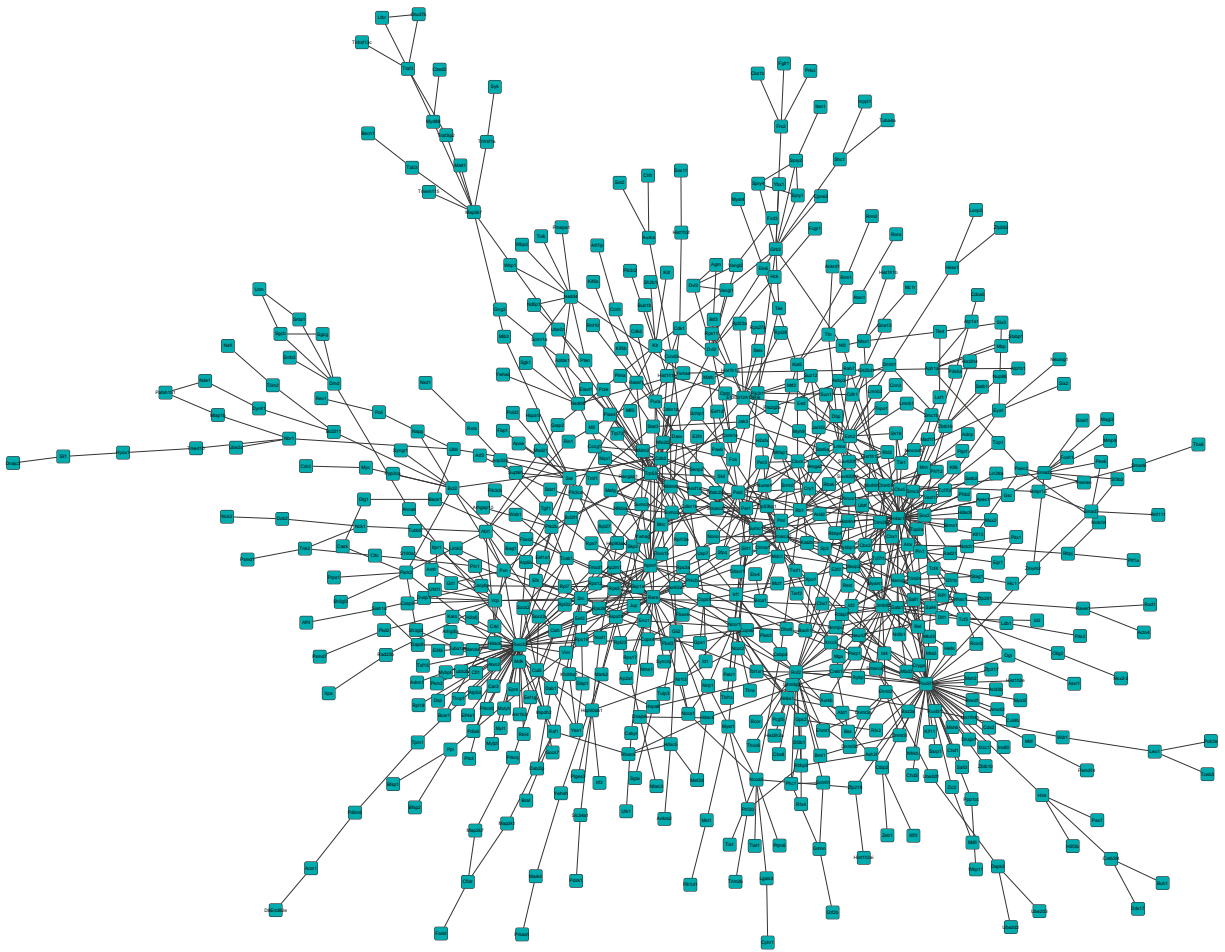

Supplement: Figure S8 — Sub-network of SOX2 in mouse BioGRID. (PDF) [file pone.0105180.s008.pdf]

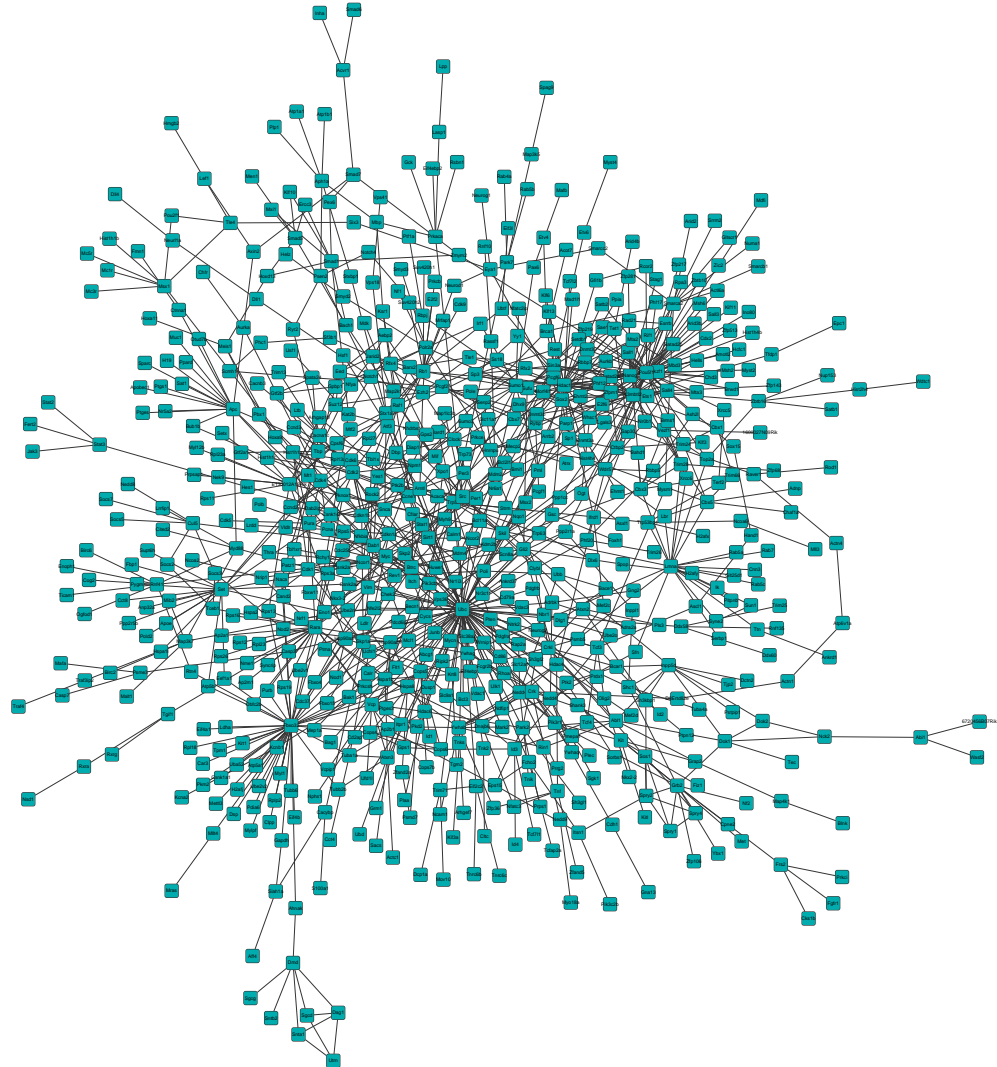

Supplement: Figure S9 — Sub-network of NANOG in mouse BioGRID. (PDF) [file pone.0105180.s009.pdf]
